# A United Nation high level meeting on chronic non-communicable diseases: utility for Africa?

**Published:** 2012-04-16

**Authors:** Justin Basile Echouffo-Tcheugui, Andre-Pascal Kengne

**Affiliations:** 1 Hubert Department of Global Health, Rollins School of Public Health, Emory University, Atlanta, Georgia, USA; 2 South African Medical Research Council and University of Cape Town, South Africa; 3 The George Institute for Global Health, Sydney, Australia

**Keywords:** United Nations, Non-communicable diseases, chronic diseases, Africa

## Editorial

Africa faces an important but seemingly “neglected epidemic” of chronic non-communicable diseases (NDCs). In many countries in this part of the world, morbidity and mortality related to NCDs such as diabetes, hypertension, ischemic heart disease and stroke have alarmingly increased over the last two decades [1–3]. Various segments of the populations are affected, but mostly young adults in urban areas. The Africa′s chronic diseases burden has been attributed to changing health-related behaviors (e.g., sedentary lifestyles and diets high in saturated fat and sugar), which are linked to the epidemiological and nutritional transitions, with structural changes such as industrialization, urbanization and increasing food market globalization [4]. The African context is compounded by weak health systems, which are unable to cope with the looming double burden of infectious and NCDs. Many have recommended an all-encompassing approach to dealing with the burden, which includes epidemiological surveillance; primordial and primary prevention (preventing disease in healthy populations); and secondary prevention (preventing complications and improving quality of life in affected populations). However, achieving these objectives confronts many challenges, which are mainly structural, logistic, human (lack of clinical and research staff) and organizational (health system).

On September 19^th^ and 20^th^ 2011, a United Nations (UN) High-level meeting on NCDs was convened in New York to raise international awareness on the facts that premature deaths from NCDs reduces productivity, curtails economic growth, and poses a significant social challenge in most countries [5]. The delegations of UN member countries were comprised of heads of state and government, parliamentarians, ministers of foreign affairs and health, and representatives of civil society. The final political declaration of the meeting highlighted the epidemic proportions of NCDs and their socio-economic and developmental impacts; the need to respond to this challenge through a whole-of-government and whole-of-society effort; the approaches to reducing risk factors and create health-promoting environments; the necessity to strengthen national policies and health systems, and the importance of international cooperation, including collaborative partnerships; as well as of research and development, and monitoring and evaluation.

A few months after the United Nations (UN) High-Level Meeting on NCDs, with the benefit of hindsight, we discuss how such a gathering may or may not help African countries to address chronic non-communicable diseases.

## How can the UN Summit positively impact on chronic non communicable diseases prevention and control in African countries?

### Increasing the awareness of chronic diseases among decision-makers and thus influence policy formulation

A UN summit can help to bring NCDs on the political agenda in African countries, especially within a context where the awareness for such issues is very low in the vast majority of countries. Local political leaders have generally not shown interest in NCDs, and this has been reflected in the budget allocation. Consequently, African ministries/departments of health are faced with a daunting task: to rally support for chronic disease prevention and control; to provide a unifying vision and action plan to ensure that inter-sectorial action is emphasized at all stages of policy formulation and implementation; and to make certain that actions at all levels and by all sectors are mutually supportive. Additionally, actions need to be prioritized in keeping with the specific population needs for NCDs prevention and control, range of possible interventions, and availability of human and financial resources to implement them. The momentum created by the UN meeting, can potentially lead to more fruitful international collaboration, coordinated by the various international organizations engaged in the prevention and control of NCDs. This will definitely help to create a framework to assist ministries of health in balancing diverse needs and priorities while implementing evidence-based interventions. A framework that will be guided by a set of principles based on a public health approach to NCDs prevention and control, and involving several levels - a national or governmental level, an inter-sectorial collaborations, as well as population-based and individual interventions. International collaborations can be beneficial for most African countries, which do not have the resources to immediately implement the needed policies to curtail NCDs. Countries would be helped to define the priorities, thus select and implement activities that are immediately feasible and likely to have the greatest impact for the investment. For example, the World Health Organization and the International Diabetes Federation have already been helping countries to formulate policies for prevention and control for NCDs [6, 7]. Such initiatives are likely to be reinforced and extended to a greater number of countries as a result of the UN summit on NCDs.

### Bringing attention to special neglected and understudied diseases – inclusion in the agenda

Hitherto, the discourse on chronic non-communicable diseases in Africa has tended to focus on cardio-metabolic disorders, including cardiovascular diseases, diabetes and obesity. Such an inclination may not be justified as many of the risk factors (e.g., physical inactivity, unhealthy diet, and obesity) are also shared by conditions like cancers. Also, given the increase in smoking rates in African countries, chronic obstructive pulmonary disease (COPD) are likely of concern. The UN summit may help to bring attention on the latters, but also other conditions like mental diseases. Indeed, cancers, COPD and mental diseases have been increasingly recognized as a major global health problem with a rising incidence and morbidity [8–10], but a limited number of studies on these conditions have been conducted in Africa. Furthermore, there is a need to scale-up health services in these countries to address the growing burden of these conditions.

## What will a UN summit not affect?

### Economic environment

A UN summit focused solely on health cannot by itself change the economic context, which has an impact on population health. In fact, globalization of the economy is one of the driving forces that spur the increase in chronic non-communicable diseases, through the introduction of processed foods and sodas in African countries. The adverse effects of trade liberalization and trade agreements on health do not seem to be avoidable on the short-term [11]. Reforms of the world trade regimes would require much greater political capital and involvement of Western countries than that observed within the framework of an UN summit solely dedicated to health.

### Lack of clinical resources and training of medical practitioners

Human resources are the crucial core of a viable health system in Africa. Hitherto, these have been a neglected component of health-system development. These include the low training capacity in Africa (two- thirds of countries have only one medical school, and some have none [12]), the decline in the investment in educational infrastructure and educators, the focus on training highly skilled but easily exportable workers, the emphasis on initial training to the detriment of continued professional development, the relatively low quality of the quality and productivity of the health workforce, professional bodies not really protecting the interests of their members, as well as poor working conditions and remuneration. All these are long-standing structural problems with local specificities that have hardly been addressed by the existing international collaborations, let alone a single UN summit.

### Research

To improve the burdensome health issues related to NCDs, African public policymakers will need to find the best solutions and the best ways to fit these solutions into complex and often overstretched and under-resourced health systems, and the best ways to bring about the desired changes in health systems. Even with a lot of good will, donors and international agencies cannot come–up with country-congruent policies that are locally applicability. Thus, country-based public health research and clinical evidence are needed for the design of appropriate policies. Otherwise, cost-effective public health interventions to tackle NCDs will not reach populations in Africa, as non-evidence based programs would more likely be patchy, low quality, inequitable, and short-lived. Interventions to be used have to account for the epidemiological profile of African population in various countries, and effective delivery mechanisms have to be context-specific as uniform approaches are bound to failure. Although the UN summit can help to improve the availability of international funds for NCDs research, a local dynamic internal to African countries is necessary to generate evidence that would serve for public health planning. Researching on NCDs is a long–term and tenuous undertaking that requires a critical mass of local researchers with sufficient expertise, which cannot be the result of a one and off external event.

## Competing interests

The authors declare no competing interests.
